# Typhoid fever: clinical presentation and associated factors in febrile patients visiting Shashemene Referral Hospital, southern Ethiopia

**DOI:** 10.1186/s13104-018-3713-y

**Published:** 2018-08-22

**Authors:** Limenih Habte, Endale Tadesse, Getachew Ferede, Anteneh Amsalu

**Affiliations:** 1 Shashemene Referral Hospital, Oromia Region, Shashemene, Ethiopia; 2 Department of Medical Laboratory Sciences, Hawassa University, Hawassa, Ethiopia; 3 Department of Medical Microbiology, University of Gondar, P.O.Box 196, Gondar, Ethiopia

**Keywords:** Blood culture, Typhoid fever, Prevalence, Ethiopia

## Abstract

**Objective:**

Although typhoid fever is a major public health problem in Ethiopia, data is not available in the study area. Therefore, this study aimed to determine the prevalence, clinical presentation at the time of diagnosis and associated factors of typhoid fever among febrile patients visiting Shashemene Referral Hospital, southern Ethiopia. A cross-sectional study was conducted from January 1, 2016, to October 30, 2016. Socio-demographic and clinical data were collected using a structured questionnaire. A blood sample was collected and inoculated into Tryptic soy broth.

**Results:**

A total of 421 adult febrile patients suspected of typhoid fever were included in the study. Of these, the overall prevalence of culture-confirmed typhoid fever was 5.0% (21/421). The prevalence of typhoid fever was significantly associated with rural residence (8.4%). As compared to the urban resident, the rural resident was 3.6 times more likely found to have culture-confirmed typhoid fever. The prevalence of typhoid fever was significantly associated with those patients whose water source was spring 7 (12.3%) and river 7 (13.2%). All of those study participants who used treated water were culture negative. Fever for ≥ 5 days, abdominal pain, and skin rash independently predicted blood culture-confirmed typhoid fever.

## Introduction

Typhoid fever is a major public health problem in low-income and middle-income countries (LMICs) like Ethiopia where there are substandard hygiene and unsafe drinking water supplies and the quality of life is poor [1–5]. Typhoid fever is a systemic infection caused by human-specific food and water-borne pathogens, such as *Salmonella enterica* subspecies, enterica serovar typhi (*S. typhi*) or by the related but less virulent *Salmonella paratyphi* A, B, and C, collectively called typhoidal *Salmonella* [6]. It is transmitted by the fecal–oral route through contaminated water [4] and food [2, 7]. In 2010, the estimated global episodes of typhoid fever ranged from 13.9 to 26.9 million cases [8]. In the same year, the estimated number of typhoid fever cases in LMICs after adjusting for water-related risk was 11.9 million cases with 129, 000 deaths that suggested a higher incidence in Africa [1]. Although national surveillance studies are lacking in Ethiopia, the individual study reported 4.1% [9] prevalence of typhoid fever among patients with febrile illnesses. Moreover, a systematic and meta-analysis study in Ethiopia showed typhoidal *Salmonella* (*S. typhi*) accounted for 42.1% of the total isolates of *Salmonella* species reported from 1974 to 2006 years indicating typhoid fever is endemic in Ethiopia [10].

The clinical presentation of typhoid fever varies from a mild illness with low grade fever, headache, fatigue, malaise, loss of appetite, cough, constipation and skin rash or rose spots to in some cases, a fatal complications such as intestinal perforations, gastrointestinal hemorrhages, encephalitis and cranial neuritis [11, 12]. Despite its clinical importance, laboratory diagnosis of typhoid fever in resource-limited countries widely depends on non-specific clinical presentation and Widal test which has a low specificity and positive predictive value (PPV) [9]. Hence, leads to wrong diagnoses and over antibiotic prescription [13] that further increases the emergence of multidrug-resistant strains for commonly used drugs [14]. However, diagnosis of typhoid fever using blood culture is the mainstay of diagnosis and has good specificity and can proceed to antibiotic sensitivity test [15]. Yet, blood culture has its own limitation; such as long turnaround time, expensive and needs microbiology experts, due to this it is not routinely performed in resource-limited countries.

Considering typhoid fever is endemic in Ethiopia and the prevalence of this disease varies with demographic, environmental and climatic data; updated information regarding the epidemiology of typhoid fever in the study area may aid policy makers to design appropriate intervention strategies. So far there is no data describing the epidemiology of typhoid fever in the study area. Therefore, the main objective of this study was to determine the prevalence of typhoid fever, clinical presentation at the time of diagnosis and associated factors among febrile patients visiting Shashemene Referral Hospital, southern Ethiopia.

## Main text

### Materials and methods

#### Study design, study area and study period

A hospital-based cross-sectional study was conducted in Shashemene Referral Hospital from January 1, 2016, to October 30, 2016. Shashemene is the town of West Arsi zone in Oromia regional state, Ethiopia.

#### Study population and sample size

All adult patients (age ≥ 18 years) suspected of typhoid fever who visited the hospital during the study period were invited to participate in the study. The sample size was estimated to be 384 by using single proportion formula at 95% confidence interval, assuming typhoid prevalence of 50% and 5% marginal error; by adding 10% contingency a total sample size was 422. Participants already on antibiotic treatment within 2 weeks and diagnosed with other known febrile illness were excluded from the study.

Typhoid fever suspected patients are defined as patients (axillary temperature, ≥ 38 °C) who reported having a fever for at least 3 days and headache. In addition, if there was clinical suspicion of typhoid fever by the attending senior clinical staff at the study sites.

Typhoid fever confirmed cases were defined as a patient with fever (axillary temperature, ≥ 38 °C) for at least 3 days with a laboratory-confirmed positive blood culture of *S. typhi*.

#### Data collection

##### Socio-demographic and clinical data

All adult febrile patients visiting adult outpatient department (OPD) clinic were clinically examined by the physicians and those suspected of typhoid fever were requested for blood culture. After obtaining written informed consent socio-demographic and clinical data were collected by nurses using a structured questionnaire that was validated and edited after small pilot study.

##### Blood sample collection and processing

In the laboratory, a total of 5 ml blood sample was collected aseptically using 70% alcohol and 2% tincture of iodine from a peripheral vein in each patient. Then the blood sample was dispensed into a sterile bottle containing 45 ml of Tryptic soy broth culture medium (Becton, Dickinson-USA).

##### Isolation and identification of bacteria

The inoculated bottles were incubated aerobically at 37 °C for 7 days in the Microbiology laboratory and observed for a sign of bacterial growth (turbidity, hemolysis, air bubbles or gas production and clot formation) on the daily bias for up to 7 days. Bottles which showed sign of growth were further processed by Gram stained and sub-cultured on MacConkey agar (Park Scientific Unlimited-England), at 37 °C for 24-h. The plate was then aerobically incubated for 18–24 h at 37 °C. A blood sample containing broth with no bacterial growth after 7 days were sub-cultured on blood agar before being reported as a negative result. Identification of isolates were done by colony morphology, Gram staining, and biochemical tests using Kligler iron agar (KIA)-(Becton, Dickinson-USA), Motility, Indole, Ornitine (MIO) (Park Scientific Unlimited-England), Citrate Utilization test (OXID LTD England), Urease test (Mast Group Ltd, UK) and lysine Iron agar (LIA)-(Liofilchem-Italy) test.

#### Data analysis

Data were entered and analyzed using SPSS version 20 software. Chi square and a logistic regression model were used to determine the predictors of typhoid fever. Adjusted odds ratio (AOR) with 95% CI was also computed from multivariable logistic regression adjusting for possible confounders. A p-value of less than 0.05 was considered statistically significant.

### Results

#### Socio-demographic characteristics of study participants

All 422 study participants who were suspected of typhoid fever by physicians during a study period were approached to the study. However, one study participant’s data was found incomplete due to this, a total of 421 adult febrile patients suspected of typhoid fever were included. The mean (standard deviation [SD]) of the participants’ age was 35.3 (11.3) years, ranged from 18 to 66 years. More than half of the patients 235 (55.8%) were females, 138 (32.8%) were in the age category of 31–40 years and 243 (57.7%) were an urban resident. The majority 254 (60.3%) were married, 177 (42%) farmer, 174 (41.3%) had no formal education and 333 (79.1%) had on the average monthly income of less than or equal to 1000 Ethiopian Birr (ETB) (Table 1).

**Table 1:** The prevalence of typhoid fever with respect to socio-demographic characteristics among febrile patients visiting Shashemene Referral Hospital, southern Ethiopia, 2016

Variables		Total (N = 421)	Culture positive (N = 21)	COR (95% CI)	p-value
Age (years)					
18–20		40 (9.5)	3 (7.5)	2.7 (0.58–12.67)	0.204
21–30		129 (30.6)	8 (6.2)	2.2 (0.65–7.54)	0.203
21–30		138 (32.8)	4 (2.9)	1	
41–50		74 (17.6)	3 (4.1)	1.4 (0.31–6.50)	0.655
> 50		40 (9.5)	3 (7.5)	2.7 (0.58–12.67)	0.204
Sex					
Male		186 (44.2)	9 (4.8)	1	
Female		235 (55.8)	12 (5.1)	1.1 (0.43–2.56)	0.90
Residence					
Urban		243 (57.7)	6 (2.5)	1	
Rural		178 (42.3)	15 (8.4)	3.6 (1.38–9.57)	0.009
Marital status					
Married		254 (60.3)	10 (3.9)	1	
D/W		97 (23.1)	7 (7.2)	1.9 (0.70–5.13)	0.207
Unmarried		70 (16.6)	4 (5.7)	1.5 (0.45–4.87)	0.520
Occupation					
Farmer		177 (42.0)	10 (5.6)	3.6 (0.45–28.7)	0.227
Merchant		125 (29.7)	5 (4.0)	2.5 (0.27–21.9)	0.408
Students		58 (13.8)	5 (8.6)	5.7 (0.64–50.0)	0.119
Employee		61 (14.5)	1 (1.6)	1	
Educational status					
No-formal		174 (41.3)	12 (6.9)	2.1 (0.72–6.12)	0.172
Primary		100 (23.8)	4 (4.0)	1.2 (0.31–4.52)	0.806
Secondary and above		147 (34.9))	5 (3.4)	1	
Monthly					
≤ 1000		333 (79.1)	18 (5.4)	1.6 (0.47–5.6)	0.448
Income (Birr)					
> 1000		88 (20.9)	3 (3.4)	1	

*D/W* divorce/widowed

The overall prevalence of culture-confirmed typhoid fever was 5.0% (21/421, 95% CI 2.9–7.1%). Rural residence showed a statistically significant association with the prevalence of typhoid fever (p-value < 0.05) while age, sex, marital status, occupation, educational status and monthly income were not significantly associated. Those study participants who were lived in rural areas were 3.6 times more likely found to have culture-confirmed typhoid fever (COR = 3.6, 95% CI 1.38–9.57) as compared to the urban resident. Students were 5.7 times (COR = 5.7, 95% CI 0.64–50.0) more likely to have culture-confirmed typhoid fever as compared with an employee (Table 1).

#### Clinical presentation at the time of diagnosis

Concerning their clinical presentation at the time of diagnosis, all the participants were febrile for at least 3 days and had a headache. The majority 358 (85.0%) presented with fever less than 5 days, fatigue 348 (82.7%) and loss of appetite 273 (64.8%); whereas 76 (18.1%), 120 (28.5%), 65 (15.4%), 187 (44.4%) and 8 (1.9%) presented with constipation, diarrhea, abdominal pain, cough and skin rash, respectively. Fever, abdominal pain, and skin rash showed a statistically significant association with the prevalence of typhoid fever while fatigue, loss of appetite, constipation, diarrhea, and cough were not significantly associated. Febrile patients who presented with a fever greater or equal to 5 days was 18.2 times (AOR = 18.2, 95% CI 6.45–54.5) more likely to have culture-confirmed typhoid fever as compared to patients presented with fever of less than 5 days. Febrile patients presented with abdominal pain was 3.9 times (AOR = 3.9, 95% CI 1.32–11.23) and skin rash was 11.1 times (AOR = 11.1, 95% CI 1.61–76.2) higher odds of culture-confirmed typhoid fever as compared to those who hadn’t have abdominal pain and skin rash respectively (Table 2).

**Table 2:** Clinical signs and symptoms of typhoid fever and their association with blood culture positivity among adult patients visiting Shashemene Referral Hospital, southern Ethiopia, 2016

Variable (n = 421)		Total N (%)	Culture pos N (%)	COR (95% CI)	AOR (95% CI)	P-value
Fatigue						
Yes		348 (82.7)	17 (4.9)	1.1 (0.37–3.46)		
No		73 (17.3)	4 (5.5)	1		
Fever						
< 5 days		358 (85.0)	6 (1.7)	1	1	
≥ 5 days		63 (15.0)	15 (23.8)	18.3 (6.78–49.5)*	18.2 (6.45–54.5)	0.001
Loss of appetite						
Yes		273 (64.8)	13 (4.8)	1		
No		148 (35.2)	8 (5.4)	1.1 (0.46–2.82)		
Constipation						
Yes		76 (18.1)	7 (9.2)	2.4 (0.93–6.16)		
No		345 (81.9)	14 (4.1)	1		
Diarrhea						
Yes		120 (28.5)	6 (5.0)	1.0 (0.38–2.65)		
No		301 (71.5)	15 (5.0)	1		
Abdominal pain						
Yes		65 (15.4)	8 (12.3)	3.7 (1.47–9.33)*	3.9 (1.32–11.23)	0.013
No		356 (84.6)	13 (3.7)	1		
Cough						
Yes		187 (44.4)	11 (5.9)	1.4 (0.58–3.37)		
No		234 (55.6)	10 (4.3)	1		
Skin rash						
Yes		8 (1.9)	3 (37.5)	13.2 (2.92–59.4)*	11.1 (1.61–76.2)	0.015
No		413 (98.1)	18 (4.4)	1	1	

*pos *Positive, *p-value < 0.05

#### Source of food and water

The majority of the study participants 358 (85%) usually consume homemade food and 253 (60.1%) obtained water supplies from the pipe. The analysis of the prevalence of typhoid fever showed a statistically significant association with the source of water (p-value < 0.05), while the source of food was not showed a statistically significant association. None of the study participants who used treated water was culture positive (Table 3).

**Table 3:** Sources of drinking water and food in typhoid fever suspected patients visiting Shashemene Referral Hospital from January to October 2016

Variable	Total N (%)	Culture positive N (%)	Chi square (p-value)
Source of water			*X*^*2*^= 19.6, *df *= 3 (0.0001)
Pipe	253 (60.1)	7 (2.8)	
Spring	56 (13.3)	7 (12.5)	
River	53 (12.6)	7 (13.2)	
Treated (Wuha agar)	59 (14.0)	0	
Source of food			*X*^*2*^= 0.51, *df *= 1 (0.473)
Homemade	358 (85.0)	19 (5.3)	
Hotel	63 (15.0)	2 (3.2)	

### Discussion

In this study, the prevalence of typhoid fever was 5.0% concordant with studies conducted in Central Ethiopia (4.1%) [9] among febrile patients with symptoms clinically similar to typhoid fever and outside of Ethiopia such as: in Kenya among adult patients (6.3%) [16] and Papua New Guinea among all age group (4%) [17]. However, our finding was higher than a study conducted in Mekelle, Ethiopia (1.6%) [13] and lower than a study conducted in Egypt (13.64%) [18]. The differences could be explained by the difference in a geographical location where participants in our study particularly rural residents are living close to rivers, and they used river water for drinking that might tend to have more typhoid risk [19, 20].

Although studies reported that there is no significant difference on the occurrence of typhoid between urban and rural environments [20], in this study rural residents were associated with higher risk of typhoid fever as compared to urban residents. This could be explained by suboptimal access to safe water and lack of hygienic education which was supported by the high prevalence of typhoid among farmers with no formal education. In addition, lack of toilet and/or hand washing practice after toilet, open defecation practices near to the springs and rivers and inadequate medical care are common in the rural residence that may serve as a carrier for transmission [20]. In this study, the high odds of typhoid fever among students as compared to the employee is perhaps as a result of lack of sufficient safe drinking water, toilet and water for hand washing after the toilet in the educational institutions. This probably creates a greater ‘opportunity’ for person-to-person transmission in these congregated sites.

The current study assessed the patterns of clinical presentation to help in case identification at presentation to health facilities where there is no confirmatory microbiological test and it was found that patients having a fever for greater than or equal to 5 days, abdominal pain and skin rash had a significant association with culture-confirmed typhoid fever. This is in accordance with the World Health Organization (WHO) guideline in that the most common manifestations of untreated typhoid fever in the first week of infection was stepped ladder fever pattern or insidious onset fever, skin rash, anorexia, mild cough and constipation [12].

### Conclusions

The study illustrated that typhoid fever was one of the causes of a significant amount of morbidity in rural communities. To confirm typhoid fever suspected cases using blood culture, considering patients clinical presentation such as duration of fever ≥ 5 days, abdominal pain and skin rash could decrease the time and cost associated with the diagnosis. Moreover, ensuring access to safe water and delivering health education to drink treated water particularly for rural residents could reduce typhoid fever transmission.

## Limitations

This study has some limitations in light of which results need to be interpreted. First, as a hospital-based study that enrolled participants in the outpatient department, typhoid patients that don’t seek healthcare at the Shashamane Referral Hospital were missed. Second, identification of species by anti-sera and antibiotic susceptibility test for isolated pathogen was not done. Third, access to safe water, toilet, environmental sanitation and occurrence of the outbreak was not assessed during the study period. But, the study was conducted almost throughout the year, relatively in larger sample size and use of the gold standard method for diagnosis may ensure the quality of the generated data.

## Abbreviations

AOR: adjusted odds ratio
ATCC: American Type Culture Collection
COR: crude odds ratio
LMICs: low-income and middle-income countries
OPD: outpatient department
OR: odds ratio
PPV: positive predictive value
SD: standard deviation
WHO: World Health Organization
*S. typhi*: *Salmonella typhi*
